# Exploring Treatment Protocol Adherence and Variations in Paroxysmal Supraventricular Tachycardia in the Emergency Department: A Multi-Center Cohort Study

**DOI:** 10.3390/medsci13020058

**Published:** 2025-05-09

**Authors:** Kevin Ku, Jack Healy, Christian A. Lee, Maha Khan, Kevin D. Chao, Saleh Hassan, Ching-Fang Tiffany Tzeng, Yu-Lin Hsieh, Andrew Shedd, Toral Bhakta, Dahlia Hassani, Eric H. Chou

**Affiliations:** 1 Department of Emergency Medicine, Baylor Scott and White All Saints Medical Center, Fort Worth, TX 76104, USAm.khan26@tcu.edu (M.K.);; 2 Anne Burnett Marion School of Medicine at TCU, Fort Worth, TX 76104, USA; 3 Department of Emergency Medicine, St. Barnabas Hospital, Bronx, NY 10457, USA; 4 Department of Medicine, Brigham and Women’s Hospital, Boston, MA 02115, USA; 5 Department of Medicine, Harvard Medical School, Harvard University, Boston, MA 02115, USA

**Keywords:** supraventricular tachycardia (SVT), emergency department (ED), adenosine, arrhythmia management, protocol adherence, guideline deviation, emergency pharmacology

## Abstract

**Background**: Supraventricular tachycardia (SVT) is a common arrhythmia requiring prompt intervention in the emergency department (ED). Despite evidence-based guidelines recommending a stepwise approach, significant variability in clinical practice persists, particularly in adenosine dosing strategies. **Objective**: This study assessed adherence to SVT treatment protocols in the ED, focusing on the efficacy of an initial 6 mg versus 12 mg adenosine dose and the use of alternative pharmacologic agents. **Methods**: This multi-center, retrospective cohort study analyzed adult patients (≥18 years) diagnosed with stable SVT in urban EDs across North Texas between 1 January 2019, and 16 January 2022. Patients who spontaneously converted to normal sinus rhythm or presented with hemodynamically unstable SVT requiring immediate cardioversion were excluded. The primary outcome was the rate of successful conversion to sinus rhythm. Secondary outcomes included frequency of adenosine administration, deviations from 2020 AHA ACLS guidelines in SVT treatment, and risk factors associated with failure to convert to sinus rhythm following adenosine administration. **Results**: A total of 439 patients were included in the final analysis. Vagal maneuvers were attempted in 26% of cases, achieving a 31% success rate. Adenosine was used in 83% of pharmacologic interventions, with 57.5% receiving 6 mg and 42.5% receiving 12 mg as the initial dose. The 12 mg dose had a significantly higher conversion rate (54.2% vs. 40.6%, *p* = 0.03). Regression analysis identified key predictors of treatment success, including comorbidities, and baseline hemodynamics. Documentation inconsistencies, particularly regarding vagal maneuvers, were noted. **Conclusions**: In our cohort, an initial 12 mg adenosine dose was more effective than 6 mg for SVT conversion in the ED. Recognizing and addressing variations in guideline adherence can play a key role in improving patient care. Further prospective research is warranted to optimize dosing strategies and evaluate the impact of standardized protocols on clinical outcomes.

## 1. Background

Supraventricular tachycardia (SVT) is a common yet potentially life-threatening cardiac arrhythmia. SVT refers to a group of narrow-complex arrhythmias with a resting heart rate exceeding 100 beats per minute, originating above the bundle of His [1,2,3]. SVT is frequently encountered in the emergency department (ED), where patients may range from asymptomatic to presenting with complaints of palpitations, chest pain, dyspnea, syncope, or, in severe cases, hemodynamic instability [4,5,6,7]. The estimated incidence of SVT is approximately 36 per 100,000 persons per year [8]. Timely recognition and effective management are critical, as untreated SVT can lead to life-threatening arrhythmias [9,10,11,12,13]. Common comorbidities, such as hypertension, coronary artery disease, and obesity may exacerbate symptoms and complicate management strategies, emphasizing the need for individualized care [4,14].

Supraventricular tachycardia (SVT) encompasses tachyarrhythmias originating at or above the atrioventricular (AV) node and is typically characterized by a narrow QRS complex (<120 ms) and a heart rate exceeding 100 beats per minute (bpm) [14,15]. It includes a broad spectrum of electrophysiological disorders, such as inappropriate sinus tachycardia, atrial tachycardia, atrioventricular nodal reentrant tachycardia (AVNRT), and accessory pathway-mediated reentrant tachycardia [10,15]. According to the European classification, atrial flutter is also categorized as SVT [10,14]. In the emergency department (ED), where time and diagnostic resources are often limited, detailed electrophysiological differentiation of SVT subtypes is typically impractical. Consequently, SVT is commonly managed as typical AVNRT in clinical settings unless the morphology and rhythm suggest otherwise—a convention adopted throughout this article. AVNRT is the most frequently encountered SVT subtype in the ED and typically presents with ventricular rates of 160 bpm or higher [16]. This rapid rhythm can compromise hemodynamic stability by reducing effective circulation, resulting in decreased perfusion and a range of systemic symptoms [17,18].

Management of SVT includes a stepwise approach, ranging from non-invasive vagal maneuvers to pharmacologic therapy and, in unstable patients, synchronized electrical cardioversion [19]. Vagal maneuvers, such as carotid sinus massage, the Valsalva maneuver, and ice water immersion, are first-line techniques that terminate SVT by enhancing vagus tone and slowing atrioventricular (AV) conduction. When vagal maneuvers fail, pharmacologic options include beta-blockers (e.g., metoprolol, esmolol), non-dihydropyridine calcium channel blockers (e.g., verapamil, diltiazem), and adenosine, a rapid-acting class V antiarrhythmic agent.

Adenosine, which inhibits the AV node conduction and prolongs the refractory period, has a rapid onset and a short half-life (10–30 s), making it the current preferred first-line pharmacological treatment in the ED [20,21,22]. However, the medication is associated with transient but distressing adverse effects, including chest pain (83%), flushing (39.4%), and a sensation of impending doom (7%) [23]. Additionally, SVT relapse within 5 min occurs in up to 57% of cases, raising concerns about its efficacy in emergent situations [24]. Given the breadth of available management options, professional organizations have published evidence-based guidelines to optimize care [8,14,25].

Despite established treatment guidelines, variation in pharmacological treatment persists in clinical practice. Recent evidence suggests that an initial 12 mg dose may be more effective than the guideline-recommended 6 mg, demonstrating a higher success rate without increased adverse effects [26]. Given this discrepancy, a retrospective analysis of adenosine administration practices may provide valuable insights into adherence to current protocols and the frequency of deviations from guideline recommendations.

## 2. Methods

### 2.1. Study Design and Setting

This multicenter, retrospective cohort study collected data from emergency departments across Texas. The study received approval from the Baylor Scott & White Institutional Review Board (reference ID: #022-217) and was exempt from informed consent due to its retrospective nature. The research adhered to the Strengthening the Reporting of Observational Studies in Epidemiology (STROBE) guidelines and complied with the Declaration of Helsinki [27].

### 2.2. Inclsion and Exclusion Criteria

Patients aged 18 years or older diagnosed with supraventricular tachycardia (SVT) between 1 January 2019 and 16 January 2022 were included if they presented to the emergency department (ED) with stable SVT, as defined by Advanced Cardiac Life Support (ACLS) guidelines—tachycardia exceeding 100 beats per minute without hemodynamic instability or end-organ damage. Diagnosis was confirmed through 12-lead electrocardiogram (EKG) readings by Emergency Medicine Attending. Patients were identified by the ICD-10 code I45.1 from physician documentation in electronic medical records [8,25]. A team manually extracted up to 120 data points per patient, including demographics, medical history, vital signs, administered medications, laboratory values, and clinical outcomes.

Exclusion criteria included pregnancy, unclear pre-hospital documentation regarding patient care, or primary admission for trauma. Additionally, patients who spontaneously converted to sinus rhythm or presented with hemodynamically unstable SVT requiring immediate cardioversion were excluded.

### 2.3. Major Outcomes

The primary outcome was the rate of successful conversion of SVT to sinus rhythm. Secondary outcomes included the frequency of adenosine administration (6 mg vs. 12 mg), deviations from 2020 AHA ACLS guidelines in SVT treatment, and risk factors associated with failure to convert to sinus rhythm following adenosine administration [8,25]. Treatment success was defined as conversion to normal sinus rhythm within 10 min of adenosine administration, regardless of dose, with the maintenance of normal sinus rhythm for at least 30 min following conversion. Given that ACLS guidelines recommend a 1–2 min wait before escalating to 12 mg of adenosine, the chosen timeframe ensured a pragmatic evaluation of treatment effectiveness while accounting for potential documentation errors, system delays, and brief SVT relapse [8,25].

### 2.4. Statistical Analysis

Statistical analysis was conducted using R (version 1.3.1093) and Microsoft Excel 2010. Patient demographics, including age, sex, and comorbidities, were summarized using means with standard deviations for continuous variables and percentages for categorical variables. Baseline clinical parameters (e.g., blood pressure, heart rate, QRS duration, and white blood cell count) were described [14]. These variables were compared between patients who successfully converted to sinus rhythm with a 6 mg dose of adenosine, those who received alternative pharmacological treatment first, and those who received 12 mg doses.

A chi-squared analysis was performed to evaluate the efficacy of using 12 mg of adenosine as an initial dosage compared to 6 mg. A regression analysis was conducted to assess risk factors influencing successful conversion to normal sinus rhythm following a vagal maneuver and at least 6 mg of adenosine. The Hosmer–Lemeshow goodness-of-fit test was used to evaluate the ability of statistical models to describe all subgroups within the regression analysis [28]. Following the initial regression evaluating any patient who received adenosine as a first-line treatment, the data were stratified by the initial adenosine dose (6 mg vs. 12 mg) to examine the effects of different doses on conversion outcomes. Patients requiring additional doses or alternative medications were considered as not converting with the initial dose.

## 3. Results

A total of 507 patients presented to the ED with symptoms of SVT. Their baseline characteristics are summarized in Table 1. Patients who received adenosine had similar baseline characteristics across dosage groups. However, those who received alternative treatments differed significantly in age and initial heart rate. Patients treated with alternative medications were older (mean 65.6 years) compared to those receiving adenosine (57.7 years for 6 mg, 52.9 years for 12 mg). Additionally, their initial heart rate was lower (median 131 BPM) compared to those treated with adenosine (165 BPM for 6 mg, 170 BPM for 12 mg).

**Figure 1:**
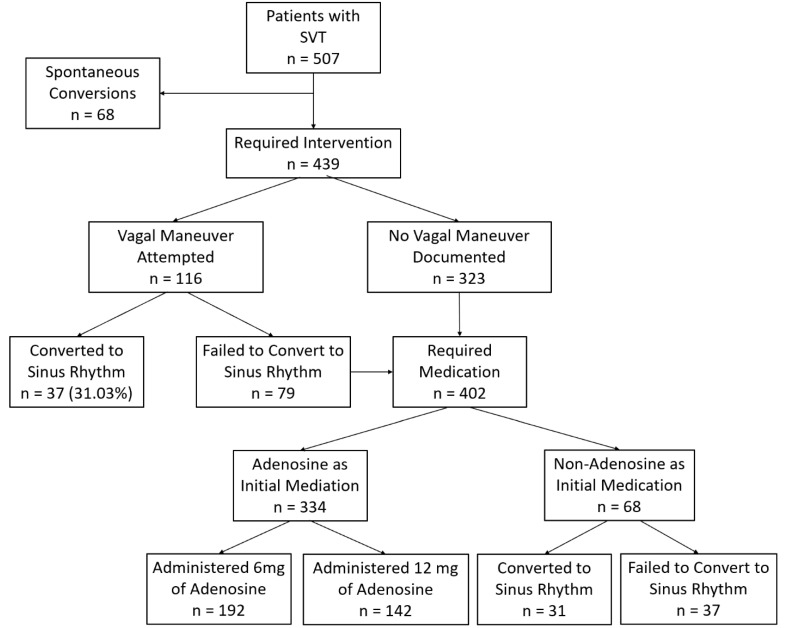
Study flow diagram. A total of 507 patients presented to the ED suspecting of SVT. This figure shows initial treatment pathways and early outcomes stratified by adenosine dose (6 mg, 12 mg, or none). For subsequent treatment steps and final outcomes in non-responders, see Figure 2.

Among these patients, 68 spontaneously converted to sinus rhythm before intervention. Of the remaining 439 cases, vagal maneuvers were attempted in 116 patients, successfully converting 37 (31.0%). The remaining 402 required pharmacological intervention. As a first-line treatment, physicians administered 6 mg adenosine in 192 cases (47.8%), 12 mg in 142 cases (34.3%), and alternative medications in 67 cases (16.9%) (Figure 1). Exploratory analysis showed that 95% of patients who successfully converted did so within 6.5 min, reinforcing the 10 min threshold as a clinically relevant measure of treatment success.

**Figure 2:**
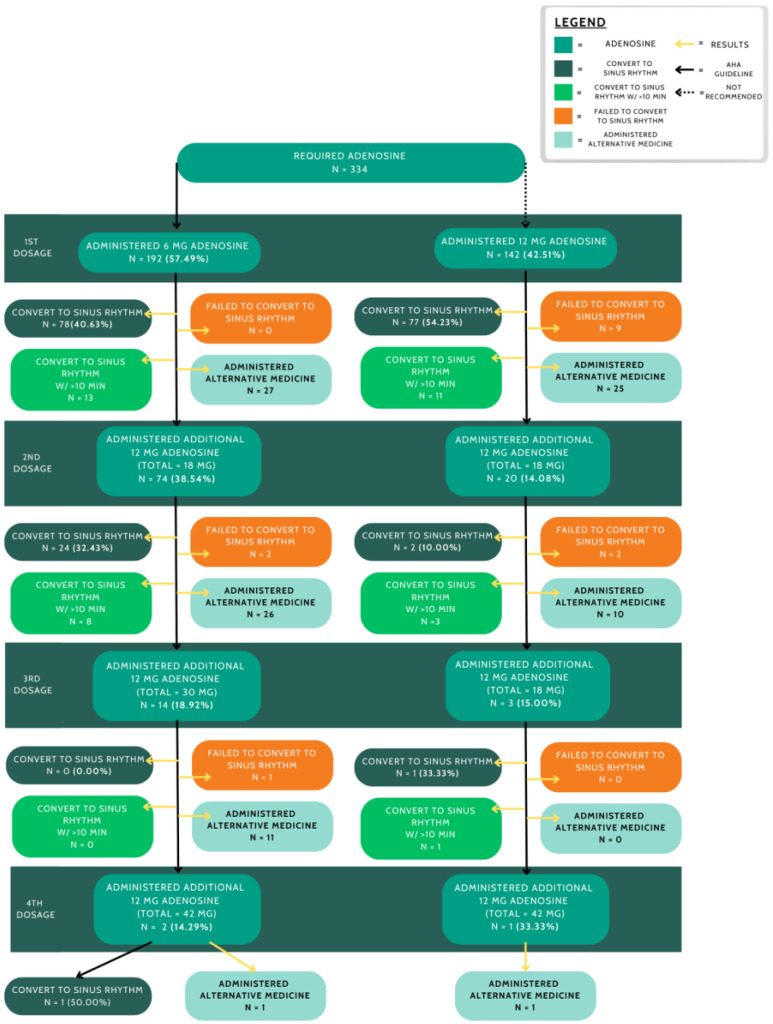
Adenosine administration with resulting outcomes. The left-hand path shows adenosine administration in accordance with the AHA guidelines, which is compared to the right-hand path, which shows the administration following 12 mg of adenosine as an initial dosage.

For patients receiving at least 6 mg of adenosine initially, 171 (42.5%) required an additional 12 mg dose (Figure 2). Alternative medications achieved a 45.6% success rate in converting patients to sinus rhythm. Furthermore, physicians administered 6 mg of adenosine as the first pharmacological treatment in 192 cases (58.7%) and 12 mg in 142 cases (41.3%). The success rate of 12 mg (54.2%) was significantly higher than that of 6 mg (40.6%) (X^2^ = 4.61, *p* = 0.03).

### Logistic Regression Analysis

A total of 238 patients were included in the regression analysis to identify risk factors for failing adenosine treatment regardless of dosage. However, the model demonstrated only 69.4% accuracy, with an AUC of 0.7. Furthermore, the Hosmer–Lemeshow test (*p* = 1.96 × 10⁻^12^) indicated poor calibration across all subgroups. Thus, our analysis focused solely on patients who received an initial dose of 12 mg adenosine.

For patients who received a 12 mg dose, the logistic regression model identified several significant predictors of successful conversion to sinus rhythm (Table 2). Tobacco use (*p* = 0.04), history of cancer (*p* = 0.02), and higher serum potassium levels (*p* < 0.001) were associated with an increased likelihood of conversion following adenosine administration. Conversely, patients with congestive heart failure (*p* = 0.02), chronic obstructive pulmonary disease (*p* = 0.009), CVA (*p* < 0.01), cirrhosis (*p* < 0.01), and chronic kidney disease (*p* = 0.01) demonstrated a decreased likelihood of conversion.

Interaction analyses revealed that the combination of tobacco use and coronary artery disease (*p* = 0.006) and diabetes with coronary artery disease (*p* = 0.007) were associated with a reduced likelihood of conversion. The final model exhibited strong predictive performance, achieving an accuracy of 81.25%, sensitivity of 77.78%, specificity of 82.61%, and an AUC of 0.87.

## 4. Discussion

Our study demonstrates that an initial 12 mg dose of adenosine is more effective in converting patients to sinus rhythm than the guideline-recommended 6 mg dose. Additionally, we identified significant deviations from standard treatment protocols, inconsistencies in documentation, and potential areas for improvement in clinical practice.

### 4.1. Vagal Maneuvers and Documentation

Despite guidelines recommending vagal maneuvers as the first-line intervention for SVT, documentation indicated that they were attempted in only 26% of cases. This low rate raises concerns about either non-adherence to guidelines or incomplete documentation. An explanation for the lack of documentation may be due to the fast-paced ED environment, which may have contributed to underreporting, as these interventions are often performed by nursing staff or in urgent scenarios where documentation may be deprioritized.

To improve adherence and documentation, standardized electronic health record (EHR) templates, such as pre-configured dot phrases in Epic, could facilitate consistent reporting. Further education and policy adaptations emphasizing the importance of recording vagal maneuvers may enhance compliance with best practices before initiating pharmacologic treatment.

### 4.2. Adenosine Dosage and Treatment Efficacy

Adenosine was used as the first-line pharmacologic treatment in 83% of cases, with 57.5% receiving an initial 6 mg dose and 42.5% receiving 12 mg. Our findings align with prior studies demonstrating superior efficacy of a 12 mg initial dose compared to 6 mg [29]. Notably, Belhassen et al. reported a 50% increase in efficacy with an initial 12 mg dose, supporting its use to improve conversion rates while minimizing repeated sinus pauses from stepwise dosing [26].

Our conversion rates (40.6% for 6 mg and 54.2% for 12 mg) were lower than the 69–91.4% rates reported in other studies [24,29,30,31,32,33,34,35,36]. Several factors could explain this discrepancy. Potential explanations include differences in definition of successful conversion to conversion or institutional factors. Currently, no universally agreed-upon time threshold exists to determine when adenosine should be deemed a failure. If conversion was assessed prematurely in our study, successful cases may have been undercounted. However, as stated in the method section of this paper, adenosine has a half-life of less than 10 s, meaning that 1 min should account for its efficacy [37]. Additionally, prior research, including one performed by the pharmacology department of Baylor Scott & White also reported lower conversion rates, suggesting that patient characteristics unique to our cohort may contribute to a higher-than-expected failure rate [26,38,39].

Additionally, prior caffeine consumption—an established inhibitor of adenosine efficacy—was not accounted for in our analysis. A study conducted by Cabalag et al. suggests that patients consuming caffeine within four hours may require a higher initial dose, reinforcing the potential benefits of defaulting to 12 mg in ED settings where such history may be unclear [40].

### 4.3. Non-Adenosine Pharmacological Treatments

Although non-dihydropyridine calcium channel blockers (CCBs), such as verapamil and diltiazem, are recommended as second-line therapies, 16.9% of patients received them as first-line treatment. While our study did not capture physician rationale, prior research suggests that some clinicians opt for CCBs based on patient history, prior adverse experiences with adenosine, or specific SVT subtypes. Given their comparable efficacy to adenosine, further investigation into physician decision making and patient outcomes with CCBs as initial therapy is warranted [24,29,30,31,41,42].

### 4.4. Key Predictors and Clinical Implications

The multivariate regression analysis or patients that received adenosine regardless of dosage demonstrated that the regression model could not evaluate all subgroups within the analysis, indicating that administration of different dosages may face distinct risk factors for treatment failure. Patients receiving 6 mg had a negative association with tobacco use and a significant association between prolonged QRS duration and treatment failure. Chronic nicotine exposure may upregulate adenosine receptors, potentially enhancing its effects, warranting further research into tailored dosing strategies [20,43,44].

For patients receiving an initial 12 mg dose of adenosine, tobacco use, malignancy, and hyperkalemia were associated with successful conversion, whereas a history of congestive heart failure, COPD, cirrhosis, and chronic kidney disease was associated with reduced efficacy. Additionally, diabetes in combination with hypertension or CAD negatively impacted conversion, suggesting a ceiling effect. The findings may be explained by increased adenosine receptor expression in diabetes, hypertension, and CAD [45,46]. On the contrary, patients with COPD, cirrhosis, and CKD might have been more likely to fail adenosine treatment due to chronically decreased adenosine clearance, leading to decreased adenosine receptor expression [47,48,49]. A case report by Chen et al. hypothesized that a different mechanism increasing the failure of adenosine risk was decreased venous return, which could also explain the findings in our study [50].

## 5. Study Limitations

Several limitations should be acknowledged. First, this study was retrospective, which inherently limits our ability to establish causality or draw definitive conclusions about the relationship between treatment protocols and patient outcomes. While causality cannot be established, our study provides important real-world insights into current practices in the ED. The reliance on existing medical records for data collection also restricts our ability to control for all potential confounders. Second, the impact of caffeine consumption, a known confounder in adenosine metabolism, was not assessed. Caffeine can alter the efficacy of adenosine by affecting its pharmacokinetics, and without accounting for caffeine intake, we cannot rule out its role in the treatment variations observed. While we did not assess this factor, our study’s focus on treatment variations and protocol adherence still provides valuable information regarding the general management of SVT in emergency departments. Third, variations in administration technique and institutional protocols could have influenced outcomes. Given that EDs across different institutions may have distinct practices for managing SVT, this variability introduces complexity in interpreting our results. However, these variations also highlight an important area for improvement in clinical practice. Fourth, due to the study design and retrospective nature, data for adverse reactions, patient disposition, or final treatment were not available for analysis. Lastly, documentation inconsistencies may have led to underreporting of vagal maneuvers, potentially affecting our findings on adherence to guideline-based care. Future prospective studies are needed to validate our findings, explore patient-specific predictors of adenosine efficacy, and assess the impact of standardizing an initial 12 mg dose.

## 6. Conclusions

Our study highlights that an initial 12 mg dose of adenosine is more effective than 6 mg in converting SVT to sinus rhythm, supporting a potential shift in first-line treatment practices. Additionally, significant gaps in vagal maneuver documentation and deviations from guideline-directed therapy were identified, emphasizing the need for improved documentation strategies and adherence reinforcement. These findings provide actionable insights for optimizing ED management of SVT and warrant further investigation into individualized treatment approaches based on patient-specific predictors.

## Figures and Tables

**Table 1:** Patient characteristics—stratified by initial treatment.

Characteristic	Total Patients Requiring Medication (*n* = 402)	6 mg Adenosine (*n* = 192)	12 mg Adenosine (*n* = 142)	Alternative Treatment (*n* = 68)
Age, mean (SD)	58.87 (15.97)	58.28 (15.36)	56.54 (16.20)	65.6 (15.92)
Male gender, *n* (%)	173 (43.03%)	78 (40.21%)	62 (43.66%)	34 (50%)
Race, *n* (%)				
Asian	9 (2.24%)	4 (2.06%)	0 (0%)	5 (7.35%)
Black	77 (19.15%)	39 (20.10%)	29 (20.42%)	9 (13.24%)
White	308 (76.62%)	148 (76.29%)	109 (76.76%)	53 (77.94%)
Other	8 (1.99%)	3 (1.55%)	4 (2.82%)	1 (1.47%)
Hispanic ethnicity, *n* (%)	50 (12.44%)	25 (12.89%)	20 (14.08%)	6 (8.82%)
Comorbidities, *n* (%)				
Diabetes mellitus	107 (26.62%)	43 (22.16%)	42 (29.58%)	23 (33.82%)
Hypertension	211 (52.49%)	93 (47.94%)	75 (52.82%)	44 (64.71%)
Smoking history	171 (42.54%)	79 (40.72%)	61 (42.96%)	31 (45.59%)
Coronary artery disease	56 (13.93%)	21 (10.82%)	25 (17.61%)	11 (16.18%)
Cancer	53 (13.18%)	23 (11.86%)	12 (8.45%)	10 (14.71%)
Initial vital signs, mean (SD)				
Systolic blood pressure (mmHg)	128.5 (27.6)	126 (35.5)	122 (35.5)	129 (30)
Diastolic blood pressure (mmHg)	83 (20)	83 (26.25)	79 (29)	81 (23)
Heart rate (bpm)	150.6 (46)	165 (66)	167 (77)	131 (73.5)
Laboratory values, mean (SD)				
Serum sodium	138 (5)	139 (5)	138 (4)	138 (4.5)
Serum potassium	3.9 (0.6)	4 (0.6)	3.9 (0.6)	3.9 (0.6)
Serum creatinine	1.07 (0.45)	1.1(0.49)	1.07 (0.46)	1.09 (0.36)
Hemoglobin	13.9 (2.8)	14.2 (2.8)	13.7 (2.7)	13.8 (1.35)
White blood cell count	9.4 (4.55)	9.8 (4.2)	9.25 (4.65)	9.2 (4.93)

**Table 2:** Logistic regression analysis for patients with an initial dose of 12 mg.

Variable	Odds Ratio (95% CI)	*p*-Value
ST EKG changes	0.51 (0.2, 1.28)	0.15
Age	1.04 (1.0, 1.07)	0.05
Smoking history	3.37 (1.09, 10.41)	0.04
DM	10.25 (0.66, 158.39)	0.10
HTN	0.65 (0.21, 2.03)	0.46
CAD	372.78 (0.54, 2.59 × 10^5^)	0.08
CHF	0.13 (0.02, 0.74)	0.02
COPD	0.08 (0.01, 0.54)	<0.01
CVA	8.05 × 10^3^ (10.13, 6.39 × 10^6^)	<0.01
Cirrhosis	0.0 (0.0, 0.09)	<0.01
CKD	0.05 (0.0, 0.5)	0.01
Dementia	0.0 (0.0, inf)	0.99
Cancer	147.53 (2.09, 1.04 × 10^4^)	0.02
Heart rate	1.01 (1.0, 1.02)	0.09
Serum BUN	0.87 (0.81, 0.93)	<0.001
Serum K	8.78 (2.76, 28.0)	<0.001

## Data Availability

The data presented in this study are available on request from the corresponding author.
